# Evaluation of a Novel Left Ventricular Assist Device for Resuscitation in an Animal Model of Ventricular Fibrillation Cardiac Arrest

**DOI:** 10.1109/JTEHM.2021.3135445

**Published:** 2021-12-13

**Authors:** Zongtao Wang, Huiming Yu, Shiping Yan, Hong Yan, Danhong Chen, Yining Dai, Qichun Xu, Zhihuan Zeng, Wei Zhang, Lijun Jin

**Affiliations:** The First Affiliated Hospital of Guangdong Pharmaceutical University Guangzhou Guangdong 510008 China; Hypertension Research Laboratory, Guangdong Provincial People’s HospitalDepartment of CardiologyGuangdong Cardiovascular Institute, Guangdong Academy of Medical Sciences Guangzhou Guangdong 510080 China; Hunan APT Medical Inc. Xiangxiang 411400 China

**Keywords:** Percutaneous implantation, left ventricular assist device, cardiac arrest, rescue

## Abstract

We evaluated an independently developed novel percutaneous implantable left ventricular assist device for resuscitation in a pig model of ventricular fibrillation cardiac arrest. The model was established in 10 domestic pigs by blocking the anterior descending coronary artery with a balloon after anesthesia. With ventilator-assisted ventilation, the independently developed percutaneous implantable left ventricular assist device was inserted via the femoral artery to assist circulation. According to whether effective circulatory support was achieved, the pigs were randomly divided into an experimental group and a control group. The experimental group was subjected to insertion of the assist device and received continuous circulatory support. The control group underwent insertion of the assist device; however, it did not start it within 15 minutes. For all animals, if successful rescue was achieved (sinus rhythm restoration within 15 minutes and maintenance for over 5 minutes), circulatory support was stopped, and the arterial blockage was removed. If sinus rhythm was not restored within 15 minutes, electric defibrillation, adrenaline injection, and removal of the arterial blockage were performed, and circulatory support was provided until sinus rhythm recovered. A determination of failed rescue was made when sinus rhythm was not restored after 1 hour. All successfully rescued animals were fed for 1 week. There were no significant differences in baseline data between the groups. All animals underwent successful novel left ventricular assist device implantation through the femoral artery. The rescue rate was significantly higher in the experimental group than in the control group (80% *vs.* 0%, $P = 0.01$). All successfully rescued animals survived after 1 week of feeding, and no eating or movement abnormalities were observed. We conclude that this independently developed percutaneous implantable left ventricular assist device can be conveniently and rapidly implanted through the femoral artery and can maintain basic circulatory perfusion during resuscitation in an animal model of cardiac arrest.

## I. Introduction

Sudden cardiac arrest is a serious complication of cardiovascular disease. It usually results from coronary heart disease, heart failure, and electrolyte disturbance. Cardiac arrest can directly lead to death. In the US, over 500,000 people develop cardiac arrest every year [1]. Although the outcome of sudden cardiac arrest has been greatly improved because of medical advances in recent years, the survival rate of patients is only 22% [2]. Besides, the majority of survivors experience certain sequelae because of irreversible neurological damage, including cerebral damage and heart-pumping failure, and partial or complete loss of self-care ability, which exerts a heavy economic burden on social medical services [2]. To decrease the disability and mortality rates of sudden cardiac arrest, resuscitation is performed to ensure effective circulatory perfusion and restore a normal heart rhythm as soon as possible [3]. Systemic perfusion is directly stopped once the heart fails to pump blood. Using an auxiliary mechanical device to replace the pump and ensure effective perfusion is an important treatment measure when autonomous heartbeats do not rapidly return to normal. An intra-aortic balloon pump (IABP) is a typical cardiac assist device that is mainly used for cardiogenic shock to reduce cardiac load in cardiogenic shock. However, in recent years, several large clinical studies have cast doubt on the clinical significance of IABP and concluded that it affords very limited flow support [4]. Meanwhile, there have been advances in research have been noted in the development of novel cardiac assist devices, such as Impella and TandemHeart, with which some success has been achieved in clinical practice as they provide much higher flow support than that affords by IABP [5]. However, the currently available left ventricular assist devices have limitations, such as complicated implantation, nonconformance to normal hemodynamics, and inability to meet complicated clinical requirements. Therefore, it is of important to develop a cardiac assist device of small size and with the capacity for rapid implantation and simple operation. Considering these needs, percutaneous implantable left ventricular assistance holds promise. Thus, in this study, we assessed a novel percutaneous implantable left ventricular assist device (Chinese invention patent number: ZL 2014 1 0468040.9. US patent number: US9981078B2) in an animal model of ventricular fibrillation cardiac arrest.

**FIGURE 1.**
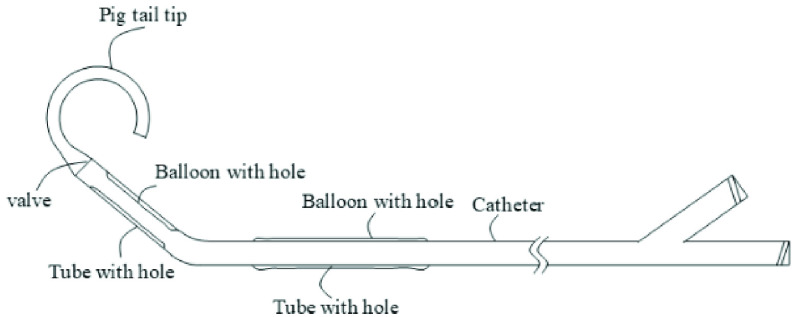
Schematic of the left ventricular assist device.

This cardiac assist device is composed of a 14-F catheter and a pumping component. The catheter consists of a ventricle portion that has a balloon with holes; it is set to be positioned in the left ventricle. The device also has an artery portion comprising a balloon with holes; it is set to be positioned in the aorta. The ventricle portion has the first inlet opening on its tube wall. The artery portion has the first outlet opening on its tube wall. One end of the catheter is closed, and the other is connected to an external drive. The pumping component, which is positioned on the ventricle portion, draws blood from the left ventricle into the catheter lumen when the external drive switches to inward pumping. The pumping component prevents luminal blood from discharging out of the ventricle portion when the external drive switches to outward pumping. The pumping component is connected to the discharge-portion balloon tube, which is covered on the outer wall of the artery portion, with both ends sealed. The balloon tube has the second outlet opening on its tube wall, which is separate from the first outlet opening. When the external drive switches to inward pumping, the discharge-portion balloon tube is in close contact with the outer wall of the artery portion. Further, when the external drive switches to outward pumping, the balloon tube and the outer wall of the artery portion separate to form a gap (FIGURE 1). Thus, blood is pumped from the left ventricle to the aorta and delivered to the whole body in a pulsatile manner. The catheter can be inserted via the femoral artery, and under the guidance of digital subtraction angiography, extended into the left ventricle across the aortic valve by examining the identification code.

The ventricular-side pumping component, positioned on the ventricle portion, draws blood from the left ventricle into the catheter lumen when the external drive switches to inward pumping. Furthermore, it prevents luminal blood from flowing out of the ventricle portion when the external drive switches to outward pumping.

The discharge-portion balloon tube, which is covered on the outer wall of the artery portion, with both ends sealed, has the second outlet openings on its tube wall. These outlets are positioned differently from the first outlet openings, and there are no overlaps. When the external drive switches to inward pumping, the discharge-portion balloon tube is in close contact with the outer wall of the artery portion. Further, when the external drive switches to outward pumping, the balloon tube and the outer wall of the artery portion separate to form a gap. The ventricular-side pumping component is a pumping-portion balloon tube, which is fixed on the inner wall of the ventricle portion, with both ends sealed. The pumping-portion balloon tube has second inlet openings, which are positioned differently from the first inlet openings, without overlaps. The pumping-portion balloon tube is made of a ductile material. When the drive switches to inward pumping, an interspace is formed between the pumping-portion balloon tube and the inner wall of the ventricle portion. When the external drive switches to outward pumping, the pumping-portion balloon tube is in close contact with the inner wall of the ventricle portion.

In this device, the ventricular-side pumping component is a unidirectional valve that is positioned inside the ventricle portion and located between the ventricle portion and the artery portion, whose direction is from the ventricle portion to the artery portion.

The ventricular-side pumping component is positioned on the catheter’s ventricle portion. The discharge-portion balloon tube covers the outer wall of the artery portion of the catheter, with both ends sealed. The heart is punctured to place the left ventricular assist device, so that the catheter’s ventricle portion is located in the left ventricle and the artery portion is located in the aorta. The two portions are separated by the aortic valve between the left ventricle and the aorta, without interfering with each other. During the operation of the device, when the external drive switches to inward pumping, negative pressure is created in the catheter. Under negative pressure, the discharge-portion balloon tube is adsorbed and is in close contact with the outer wall of the artery portion. As the second outlet openings have different positions from the first outlet openings, without overlaps, the discharge-portion balloon tube seals the first outlet openings on the artery portion, so that the catheter’s artery portion does not communicate with the aorta. At the same time, under the inward pumping of the external drive, the ventricular-side pumping component draws blood from the left ventricle to the catheter’s ventricular side and into its whole lumen. Thereafter, when the external drive switches to outward pumping and pressurizes the catheter, the ventricular-side pumping component prevents the blood in the catheter from flowing to the left ventricle from the ventricle portion. At the same time, under positive pressure, the discharge-portion balloon tube is released from the close fitting with the artery portion. An interspace for communication is formed between them, so that the first outlet openings communicate with the second outlet opening, and the blood enters into the aorta from the artery portion of the catheter. Via the inward and outward pumping of the external drive, a cycle is formed, pumping the blood from the left ventricle to the aorta. The left ventricular assist device has a simple structure and does not need to be provided with artificial lungs and other components. It will not stop working in case of pulmonary circulation interruption, indicating enhanced reliability. Furthermore, as only a puncture for its application, it is less traumatic to the body tissues and does not damage blood cells.

## II. Materials and Methods

### A. Establishing a Model of Sudden Cardiac Arrest

In a clinical setting, sudden cardiac arrest usually develops from ventricular fibrillation, which causes hemodynamic disturbance and eventually ventricular standstill. The sudden cardiac arrest model in this study was established by blocking the left anterior descending artery. The model was considered to have been successfully established herein if any of the following conditions occurred in the experimental animals: ventricular fibrillation, electromechanical dissociation, a flatline on an electrocardiogram, or a flat arterial pressure waveform [6].

The experimental animals were fasted for 24 hours and administered ketamine to induce anesthesia followed by continuous inhalational anesthesia with ether. After successful anesthesia, the animals underwent electrocardiography and blood oxygen saturation monitoring. The left femoral artery was punctured to insert a 6-F sheath, along which the guide catheter was sent to the coronary ostia for coronary angiography. A suitable balloon was selected to block the proximal and middle segments of the anterior descending coronary artery to induce acute myocardial infarction. The distal arteries were not visualized on coronary angiography.

### B. Left Ventricular Assist Device Implantation and Monitoring System

The right femoral artery was punctured to place a 6-F femoral sheath, and a long guide wire was passed to the ascending aorta. Then, the femoral sheath was removed, and a 16-F delivery sheath was inserted. Through the delivery sheath, a 14-F catheter-mounted axial pump was sent into the left ventricle under the guidance of digital subtraction angiography.

The carotid artery was punctured to place a 6-F sheath, which was connected to a pressure transducer to monitor the pressure in the carotid artery. The monitoring system(Mindary, China, Shenzhen) can record the blood pressure, the heart rate and blood oxygen concentration.

### C. Data Recording

**FIGURE 2.**
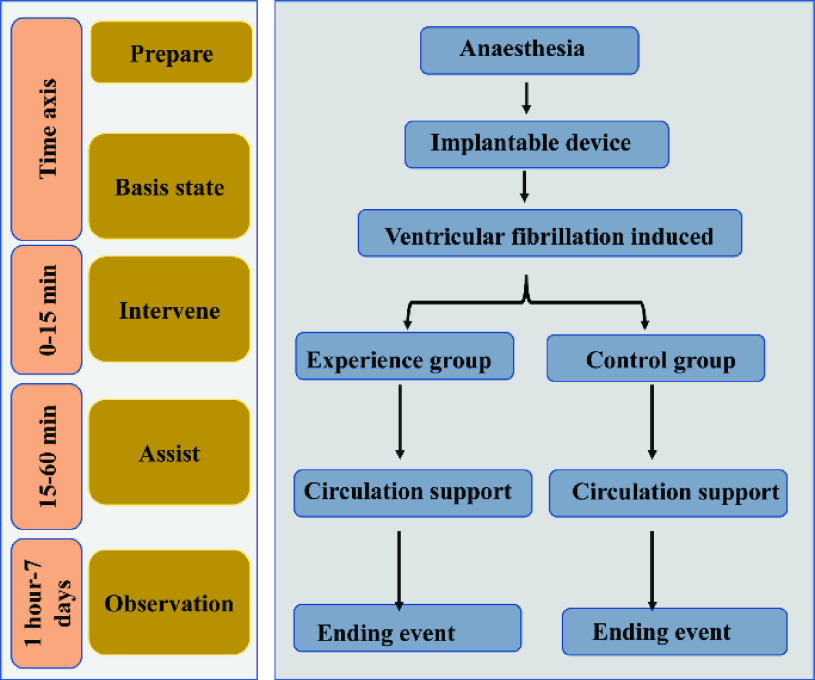
After successful model establishment, the experimental group immediately received pump support until recovery of an autonomous heartbeat. In the control group, pump support was not stated for 15 minutes, and if an autonomous heartbeat was not recovered after 15 minutes, the animals underwent pump support, adrenaline injection, electric defibrillation, and removal of the coronary artery blockage. Rescue was determined to have failed if an autonomous rhythm was not restored after 1 hour, and the experiment ended. All animals demonstrating recovery of an autonomous heartbeat were fed for one week.

Arterial pressure, heart rate, and operation time were recorded before the operation and at the time of successfully establishing a cardiac arrest model. Blood samples were collected to monitor routine blood parameters. The animals were left for one minute after successful model establishment, and the time was recorded. The animals were randomly divided into either experimental group or control groups. Then, the cardiac assist device was started in the experimental group, and the carotid pressure was recorded. The left ventricular assist device was operated with a flow rate of 2.4 L/min (stroke volume, 60 mL; 40 strokes/min). When heartbeat returned to sinus rhythm, arterial pressure, heart rate, and time were recorded. In the sham control group, the cardiac assist device was placed but not started, and arterial pressure, heart rate, and operation time were recorded. If sinus rhythm recovered within 15 minutes, the time to sinus rhythm recovery as well as the arterial pressure and heart rate were recored. Then, the blockage in the coronary artery was removed. In both groups, if sinus rhythm did not recover after 15 minutes, the arterial blockage was removed, and the time, arterial pressure, and heart rate were recorded simultaneously. Subsequently, cardiopulmonary resuscitation was performed in all animals. The experimental group continued to receive axial pump support and underwent defibrillation and intravenous adrenaline injection, while the control group underwent pump support, defibrillation, and intravenous adrenaline injection. When an autonomous rhythm was observed, resuscitation time, arterial pressure, and heart rate were recorded. All successfully rescued animals underwent routine blood examinations and were fed for one week to observe their behavior. In animals that did not recover sinus rhythm, the operation was ended after one hour of continuous pump support followed by a routine blood examination. The experimental steps are outlined in FIGURE 2.

We calculated the normalized index of hemolysis (NIH) to assess the degree of red blood cell destruction and used the change in the platelet count to assess the degree of platelet destruction. The NIH calculation formula was as follows [7]:

\begin{align*}\text {NIH} = (\Delta \text {FHB} \div \text {T}) \times &[(100 - \text {Hc}t) \div 100] \times (\text {V} \times 1000) \\&\qquad \qquad \qquad \times [1 \div (\text {Q} \times 1000)]\end{align*}

where FHB is free hemoglobin; $\Delta $FHB refers to the increment (mg/dL) in FHB during the test time interval; T is the test time interval (min); Hct is the hematocrit value (%); V is the total circulatory volume (L) (estimated by 80 mL/kg); and Q is the blood pump flow rate (L/min) (blood was pumped at a stroke volume of 60 mL and a rate of 40 strokes/min, with a flow rate of 2.4 L/min).

### D. Statistics

Any animals that died of anesthetic accident or failure to implant the device, or failed to show cardiac arrest, will be excluded. SPSS 22.0 (Version 22.0. Chicago: IBM SPSS Inc) was used for data analysis. Data are presented as mean ± standard deviation. The $t$-test was used to analyze continuous data, and Fisher’s exact test was used to analyze categorical data. A $P$ value of < 0.05 was considered statistically significant.

## III. Results

The two groups were comparable in terms of age (in months), weight, systolic blood pressure, diastolic blood pressure, heart rate, and routine blood parameters at baseline, with no significant differences (TABLE 1).

**TABLE 1:** Baseline Characteristics of the Animals Included in the Two Groups

Terms	Group		P-value
	Experimental group (n=5)	Control group (n=5)	
Sex (male%)	40	60	0.500
Weight (kg)	59.4±5.58	57.3±5.09	0.592
Time (months)	<114±0.54	11.6±1.34	0.766
Systolic pressure (mmHg)	118±16.26	122±17.91	0.736
Diastolic pressure (mmHg)	80±13.39	83±18.97	0.816
Heart rate (bpm)	85±15.18	94±14.05	0.277
Modeling time (min)	26±10	21±7	0.476
Preoperative erythrocyte count (*10^12/L)	6.37±0.57	6.16±0.23	0.518
Preoperative hemoglobin (g/L)	109.4±10.92	110.5±5.74	0.862
Preoperative leukocyte count (*10^9/L)	19.52±19.98	17.75±8.26	0.771
Preoperative platelet count (*10^9/L)	339.6±85.51	351.0±69.55	0.836

**FIGURE 3.**
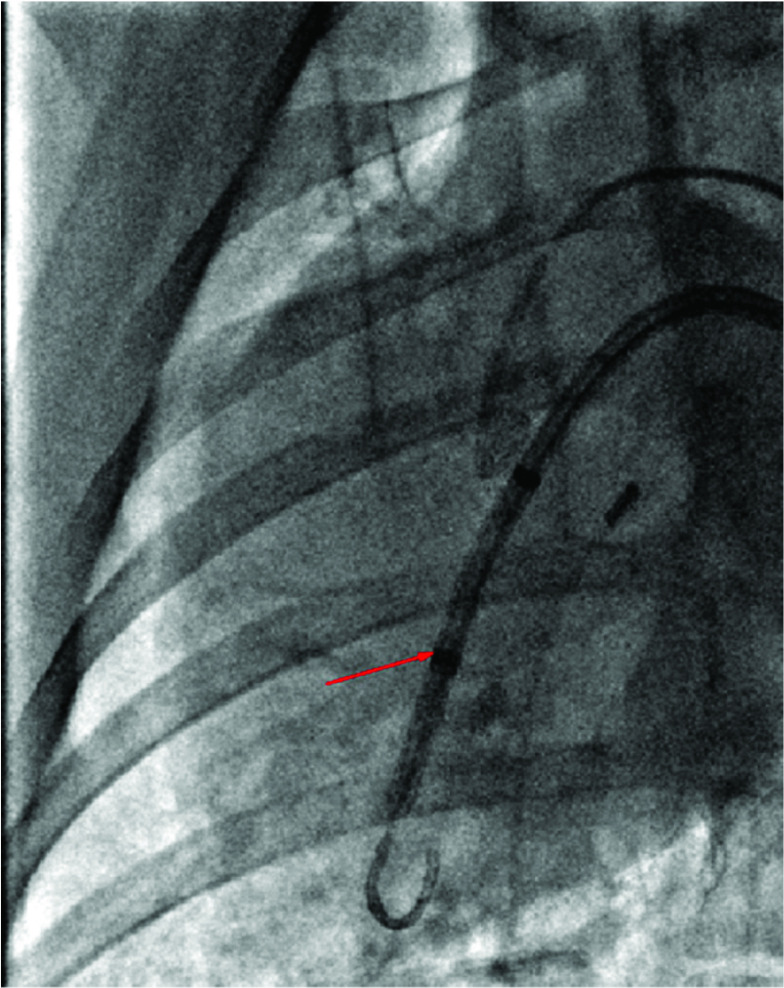
The left ventricular assist device was successfully placed into the heart, with the inlet opening of the axial pump located in the left ventricle and the outlet opening in the aorta (indicated by arrows).

**FIGURE 4.**
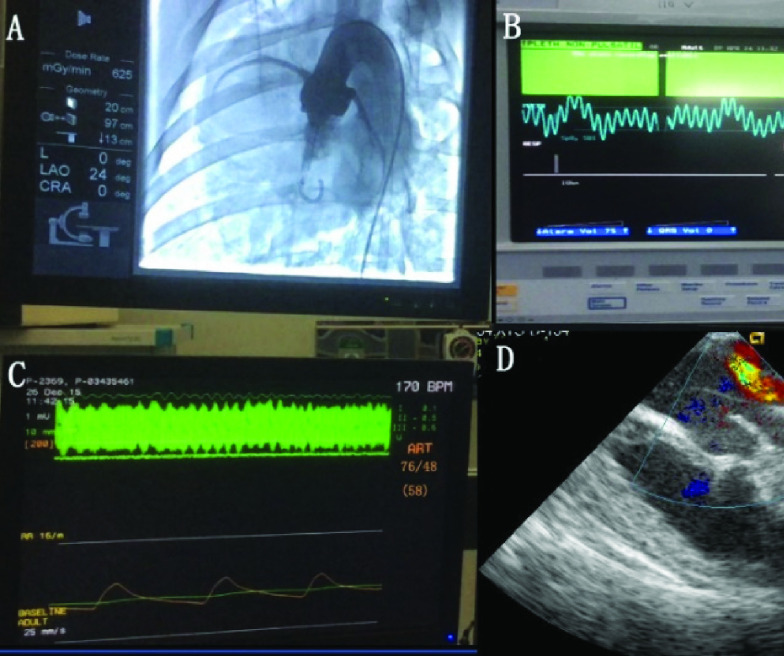
After starting the left ventricular assist device, blood was pumped from the left ventricle to the ascending aorta, with good coronary artery filling (a). Blood oxygen saturation at the peripheral body surface was 100% (b). The pressure in the carotid artery was 76/48 mmHg (c). No serious aortic valve reflux was observed on color Doppler imaging (d).

The left ventricular assist device was smoothly implanted through the femoral artery in all animals. The mean implantation time did not differ significantly between the experimental group and the control groups (5.4 ± 0.9 min *vs.* 5.2 ± 2.1 min, $P = 0.886$). The cardiac pump was successfully delivered to the left ventricle, straddling the aortic valve (FIGURE 3), with the inlet opening of the catheter positioned in the left ventricle and the outlet opening positioned in the ascending aorta. When the axial pump was started, the coronary arteries were filled and well visualized. Ultrasonography showed blood flow in the aorta, and there was no significant valve reflux on color Doppler imaging (FIGURE 4). The process of insertion was smooth, and did not involve catheter breakage or arterial injury. No thrombosis or pump seizure was noted during the cardiac device operation.

After rescue with the left ventricular assist device, four animals survived and one died in the experimental group. All five animals died in the control group. The experimental group showed a significantly higher rate of successful rescue in comparison with the control group (80% *vs.* 0%, $P = 0.01$). The four surviving animals were successfully fed for one week. They showed normal behaviors when eating and performing activities; no abnormal behavior was observed. In animals receiving pump support, peripheral blood oxygen saturation was relatively stable and could be maintained at 100%. The cardiac device was successfully operated in all animals, and there were no adverse events, such as bleeding or pump seizure, during cardiac device operation. No severe hemolysis occurred. The NIH showed no significant difference between the two groups, and the mean NIH value of all animals was 0.032 ± 0.012 g/100 L.

## IV. Discussion

Cardiac arrest is defined as the loss of the pump function of the heart, and is characterized by with the absence of a detectable pulse, unresponsiveness, and the absence of normal [8]. The causes of cardiac arrest are broad, and include: acute coronary syndromes and pulmonary embolism. Ischemic events are closely related to the occurrence of cardiac arrest, especially acute coronary syndrome [9]. With the increasing prevalence of cardiovascular disease, critical cardiac events are becoming increasingly frequent, imposing a major challenge for clinical management because of the high disability and mortality rates [10], [11]. The key measures to rescue patients with sudden cardiac arrest are to ensuring effective circulatory perfusion and restoring an autonomous heartbeat [12]. When an autonomous heartbeat cannot be restored in a timely manner, maintaining effective perfusion is the only approach to rescue life.

Circulatory assist devices provide patients a chance to survive a cardiac arrest. The current left ventricular assist devices, such as the IABP, Impella, and TandemHeart, are mainly used for cardiogenic shock. They can partially replace cardiac function, reduce cardiac preload and afterload, and allow the heart time to recover. However, the shortcomings of these devices, including insufficient flow or the fact that they do not conform to the physiological features of pulsatile blood flow, limit their use in clinical practice. The IABP, with a long history of use in cardiac support, is still the most commonly used in circulatory support device, particularly in patients with acute myocardial infarction with cardiogenic shock. However, the flow rate of the IABP is relatively low; thus, it cannot provide adequate systemic perfusion in cases of complete cardiac cessation. The NRMI-II research [13] is a large AMI registry study that evaluated the efficacy of IABP to reduce the mortality of hospital to ST-elevation myocardial infarction complicated by cardiogenic shock treated with primary angioplasty. The results demonstrated that patients with acute ST-elevation myocardial infarction with cardiogenic shock cannot benefit from routine IABP support. The IABP-SHOCK II study also questioned the clinical value of IABP.

Impella 2.5, which is a novel percutaneous implantable left ventricular assist device, offers the advantages of simple implantation and independence from native cardiac pulsation. It has been applied in the treatment of acute myocardial infarction and end-stage heart failure. The PROTECT I and PROTECT II studies [14], [15] supported the value of Impella 2.5 in cardiogenic shock and complex interventions, but suggested that it offers no significant clinical benefit in terms of mortality. TandemHeart is an extracorporeal centrifugal cardiac pump characterized by high flow and portability. It can greatly increase ejection fraction, improve renal function, and decrease myocardial oxygen consumption, but it does not offer leading survival benefits [16]. Moreover, in the real-world analyses, Impella does not offer leading benefits over IABP [17]. Although the flow-related advantages of continuous-flow cardiac assist devices, such as Impella and Tandemheart cannot be denied, pulsatile devices represented by IABP have always been available. The key is to provide pulsatile circulatory support in line with the characteristics of human physiology and ensure effective perfusion of the heart, brain and kidney [18].

The percutaneous pulsatile implantable cardiac assist device in this study offers multiple advantages over its counterparts. It is small and simple to operate, and it also addresses the limitations of many devices that do not conform to physiological features. The axial pump is mounted on the catheter and delivered into the left ventricle, riding the aortic valve. Through the inlet opening in the left ventricle and the outlet opening in the ascending aorta, the device draws blood from the left ventricle to the ascending aorta to replace the lost pumping function of the heart. Powered by an extracorporeal drive, the pump works in a pulsatile manner to provide adequate perfusion to peripheral organs and tissues. This device can accomplish the most important functions of the heart: drawing blood from the left ventricle to reduce preload and pumping blood to the aorta to ensure normal perfusion of the heart, brain, and kidneys.

The results show that this newly developed left ventricular assist device could completely replace the heart when it stops beating, with a systolic blood pressure of approximately 80 mmHg, thereby maintaining perfusion of critical organs, including the heart and brain. Peripheral blood oxygen saturation monitoring revealed that peripheral blood oxygen saturation fluctuated with axial pump-driven perfusion, indicating that this device could completely replace the heart to assist circulation. Four of the five animals in the experimental group were successfully rescued and demonstrated no significant changes in eating behavior or movement in comparison with their behaviors before the experiment. This device not only provided an effective blood supply to critical organs, but it also worked in a pulsatile manner to fulfill the physiological function of the heart as a pump. However, we did not use hypothermia treatment after cardiac arrest in this study since it may have lead to a negative impact on the prognosis.

There was no severe hemolysis, bleeding, or thrombosis after continuous pumping. The NIH value was 0.032 ± 0.012 g/100 L with this device, which is similar to international counterparts [7], indicating good blood compatibility.

## V. Conclusion

This novel axial cardiac pump assists the circulation in a pulsatile manner. This study demonstrated the effectiveness of this device in a pig model of ventricular fibrillation cardiac arrest. The device can replace the pumping function of the heart to ensure basic perfusion of vital organs, such as the heart and brain, in animals. Thus, it holds promise for rescuing patients with critical cardiac diseases, such as acute or chronic heart failure and sudden cardiac arrest.

## Data Availability

The experimental data can be accessed from Dr. Zongtao Wang on a request.
